# Allogenic Cancellous Bone versus Injectable Bone Substitute for Endoscopic Treatment of Simple Bone Cyst and Intraosseous Lipoma of the Calcaneus and Is Intraosseous Lipoma a Developmental Stage of a Simple Bone Cyst?

**DOI:** 10.3390/jcm12134272

**Published:** 2023-06-26

**Authors:** Andreas Toepfer, Michael Strässle, Ulrich Lenze, Florian Lenze, Norbert Harrasser

**Affiliations:** 1 Orthopaedics and Traumatology, Kantonsspital St.Gallen, Rorschacher Strasse 95, CH-9007 St.Gallen, Switzerland; 2 Department of Orthopaedics and Sportorthopaedics, Klinikum rechts der Isar, Technical University Munich, Ismaninger Straße 22, 81675 München, Germanynorbert.harrasser@atos.de (N.H.); 3 ECOM Excellent Center of Medicine, 81925 München, Germany

**Keywords:** simple bone cyst, intra-osseous lipoma, lipoma of bone, calcaneal bone cyst, endoscopy, ossoscopy, benign bone tumor, allografting, injectable bone substitute, white-out

## Abstract

Simple bone cysts (SBCs) and intraosseous lipoma (IOL) of the calcaneus are rare tumor entities that are primarily diagnosed due to unspecific heel pain, incidental findings, or rarely due to pathological fractures. Compared to traditional open tumor resections, endoscopic resection of these benign tumors aims to minimize surgical morbidity and maximize surgical efficiency without compromising safety. Grafting is regularly performed to reduce the risk of recurrence and stimulate osseous consolidation of the lytic lesion. As the incidence is low and treatment strategies are heterogeneous, there is no clear consensus for the treatment of simple cysts or intraosseous lipomas of the calcaneus. The objectives of this study are (a) to present medium to long-term results after endoscopic resection and grafting with allogenic cancellous bone or bioresorbable hydroxyapatite and calcium sulfate cement, and (b) to add further evidence to the discussion of whether calcaneal SBC and IOL are the same entity at different developmental stages. Between 2012 and 2019, a total of 25 benign bone tumors consisting of 17 SBCs and 8 IOLs were treated by A.T. with endoscopic resection and grafting, comprising the largest cohort to date. For grafting, 12 patients received allogenic cancellous bone (group A) and 13 patients received injectable bone substitute (group B). Pre- and postoperative imaging using plain X-rays and MRI was retrospectively analyzed with a mean follow-up time of 24.5 months to assess tumor size, osseous consolidation (modified Neer classification), and tumor recurrence. A retrospective chart analysis focusing on adverse intra- and perioperative events and other complications associated with the surgical procedure was performed using the modified Clavien–Dindo classification (CD1-3). A total of 12/13 cases with allogenic bone grafting showed a Neer Type 1 osseous healing of the tumorous lesion after endoscopic resection, whereas only 5/11 cases with injectable bone substitute showed sufficient healing (types 1 and 2). There were three recurrent cysts (Neer 4) and two persistent cysts (Neer 3) after using injectable bone substitute. Two CD1 complications were observed in group A (prolonged wound drainage, sural neuritis) and eight complications were observed in group B (6× CD1, 2× CD3). At least two IOLs diagnosed preoperatively using MRI were ultimately identified as SBCs upon histopathologic examination. Allogenic cancellous bone grafting after endoscopic resection of calcaneal SBC or IOL showed a very low rate of complications and no tumor recurrence in our series. On the other hand, depending on the material used, injectable bone substitute showed a high rate of “white-out” (excessive drainage), resulting in multiple complications such as prolonged wound healing, insufficient permanent defect filling, recurrence, and revision surgery. Over time, calcaneal SBC may transform into IOL, exhibiting distinct features of both entities simultaneously during ossoscopy and histopathological analysis.

## 1. Introduction

According to WHO’s classification of soft tissue and bone tumors, a simple bone cyst (SBC) is an intramedullary, usually unilocular, cystic bone tumor lined by a fibrous membrane and filled with serous or serosanguineous fluid. The older term, unicameral bone cyst, is no longer recommended [1]. The occurrence of SBC in the calcaneus is considered to be relatively rare, representing 2 to 14% of all SBCs [1,2,3,4,5]. In our study on the distribution patterns of foot and ankle tumors observed at a university tumor institute between 1997 and 2015, SBC was the most common benign bone tumor, accounting for 50 (21.6%) out of 231 non-malignant bone tumors [6]. In the largest review on foot tumors to date conducted by Ruggieri et al., there were 51 calcaneal cysts in 1170 cases of foot tumors. SBC accounted for 6.5% of cases in their subgroup of benign osseous lesions of the foot (consisting of 578 benign bone tumors and 201 pseudotumoral bone lesions) [7].

SBC of the long bones most commonly occurs in children and adolescents, with a reported average age at diagnosis of 9–11 years [5,8]. On the other hand, *calcaneal* simple bone cysts are observed later, usually during the second and third decades of life [9,10,11]. In a review conducted by Levy et al. in 2015 including 10 studies comprising 171 patients with SBC, the mean age was reported as 25.7 ± 8.1 years, and males were affected in two-thirds of the cases [12]. The average age of patients with *calcaneal* SBC (*n* = 42) in our series of foot and ankle tumors was 18.1 ± 6.8. There was a male predominance, with a m:f ratio of 2.2:1 [6].

Localized in the lower extremity, SBCs may cause persistent pain and thus warrant surgical therapy. However, they are diagnosed as an incidental finding in approximately 30% of cases [13,14,15]. In addition to pain, the risk of pathological fracture is another indication for surgical treatment [14,16]. According to the criteria established by Pogoda et al., the risk of a pathological fracture is increased when the cyst occupies 100% of the mediolateral cross-section in the coronary plane and more than 30% in the sagittal plane. Even in asymptomatic cases, surgery might be indicated if the critical size is reached [17]. Rarely, surgery may also be considered due to tumor anxiety following incidental findings.

Lipoma of bone is a benign neoplasm composed of white adipocytes arising within or on the surface of bone. Intraosseous lipoma (IOL) commonly arises in the calcaneus and metaphysis of long tubular bones, especially the femur, tibia, and humerus. Approximately 70% of IOL cases involve the lower limb [1]. Intraosseous lipoma is often considered to be one of the rarest primary bone tumors [18] and may be asymptomatic (30%) or produce aching pain (70%). IOL is rare and accounts for <0.1% of primary bone tumors. The age of affected patients is reported to range from the second to eighth decades of life. Males are affected more frequently than females (m:f ratio of 1.3:1) [1]. In our assessment of 413 foot and ankle tumors, there were 21 cases of IOL, all localized in the calcaneus, making it the fifth most common benign bone tumor in our series. The mean patient age was 39.4 ± 12.4, and there were 14 male and seven female patients affected by calcaneal IOL (m:f ratio 2:1). In a publication by Radl et al. including 29 cases of IOL, the mean patient age was 48 (20–75) [19].

IOL can be misinterpreted as plantar fasciitis and other common causes of heel pain [20,21,22]. In cases where a calcaneal SBC or IOL is detected on diagnostic imaging (often performed for unspecific heel pain), surgical intervention should only be considered after other causes for heel pain have been thoroughly assessed and excluded.

**Figure 1:**
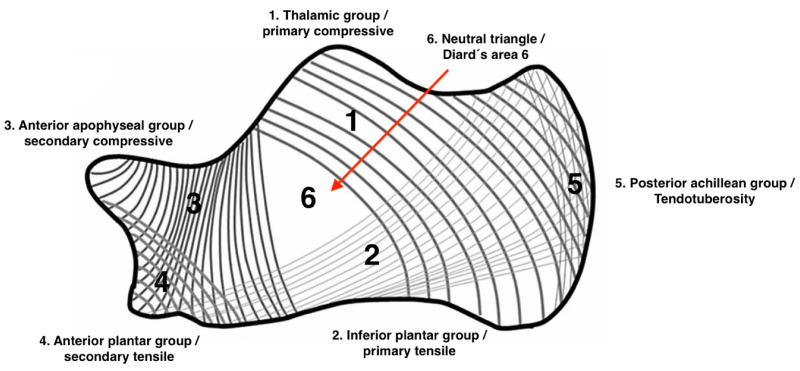
Schematic representation of the calcaneal trabecular structures according to the classification of Diard et al. [3]. The trabecular bone architecture of the calcaneus is formed by five trabecular groups, with the first three main trabecular groups defining a central area characterized by reduced bone density and increased radiolucency (Diard’s Area 6) [3,23].

SBC and IOL are similar in many ways, including their location and radiological appearance, but their content differs. SBCs contain fluid, whereas lipomas contain fat, although some lesions may exhibit a mixed content [10]. Both calcaneal SBC and IOL are almost exclusively located in Diard’s Area 6, the ventral triangular area between the major trabecular groups under the calcaneal sulcus (Figure 1), which is situated on the anteroinferior margin of the posterior articular surface [3,23,24]. Their radiological appearance is also identical, forming a triangular area that lacks a trabecular structure. It is well delineated and sometimes surrounded by a sclerotic margin that is usually thin. The defect may display signs of moderate chronic expansion, most often predominating the infero-lateral trgion, with cortical thinning and bowing (visible on tangential axial radiographs) and no periosteal reaction. This appearance has been described for both simple bone cysts and intraosseous lipomas of the calcaneus [10,17,18,25,26,27,28,29,30].

The anterior calcaneal location of SBC and IOL corresponds exactly to a triangular anatomic area in which the trabecular network is naturally much sparser (Diard’s Area 6, Figure 1). This area is visible on 70% of any normal lateral radiograph and has a pseudocystic appearance in 7% of cases. The term “*pseudocyst*” for this area of decreased radiopacity was first used by Irry et al. to describe these findings [31]. Unlike cysts and lipomas, pseudocysts contain a sparse trabecular structure and do not exhibit peripheral sclerotic rims, signs of expansion, or central calcification [32]. The term “*pseudocyst*” may be confusing in the setting of simple bone cyst as pseudocyst is not lined by fibrous membrane and is not a true SBC. The pathogenesis of the two entities, SBC and IOL, is a subject of ongoing controversy. In their 2017 publication, Malghem et al. discussed in detail the most common hypotheses regarding the pathogenesis of calcaneal SBC and IOL [10]. For SBC, various factors were discussed as possible causes, including development from a congenital remnant in the region of the primary ossification center [33], degenerative processes in areas of bone rarefaction [34], increased mechanical stress [35], organized hemorrhages [36], ischemic degeneration [25,28], and others. Additionally, the origin from ganglion cysts arising from the subtalar joint [37] or the collapse of the sinus tarsi artery [38] have been discussed. Cohen et al. indicated that the principal etiological factor of solitary bone cysts is the blockage of interstitial fluid drainage, hypothesizing that venous obstruction is the primary cause of simple bone cysts in patients with long bone cysts [39,40]. Similarly, Andermahr et al. proposed that the development of a calcaneal cyst is secondary to a disturbance in local intraosseous blood circulation [41]. Our own findings support the hypothesis of Chigira and Andermahr, which suggests a disorder in physiologic intraosseous pressure and blood circulation [41,42,43].

Regarding IOL, most authors believe that they are true benign tumors resulting from adipocyte proliferation [18,44,45]. Other authors have suggested IOL is the end result of a process of involution of other bone lesions [28,46,47,48,49]. According to Milgram, IOL may present with varying features depending on its stage of evolution. Stage 1 lesions consist of solid fat cells and demonstrate a purely radiolucent lesion with resorption of the preexisting bone. Stage 2 lesions demonstrate similar features, but also contain localized regions of increased roentgenographic density due to calcified fat. Stage 3 lesions are characterized by reactive ossification around the calcified fat in the outer rim of the lesions. Moreover, many of Stage 3 lesions contain cystic regions [18].

As stated by Malghem et al., the discussion of the pathogenesis of these two entities is confusing, especially because calcaneal cysts have been described as lipomas and vice versa. Lagier et al. believe that lipomas and cysts of the calcaneus may constitute two types of responses to a single mechanical or vascular stimulus [47], while Mirra even uses the two terms to describe a single entity with a varying cyst- or lipoma-like histology [2]. Thus, the possibility that the one may evolve into the other has been considered in both directions [10,30].

Previously, several authors have described a potential continuum between calcaneal cysts and lipomas, demonstrating through long-term MRI follow-ups that SBC can heal with fatty conversion of the cystic cavity, resulting in partly cystic remnants [10,30,50]. Thus, it has been proposed that at least part of the so-called intraosseous lipomas are actually healed simple bone cysts [50].

In our case series, the combination of preoperative MRI, intraoperative high-resolution endoscopic imaging of the lesion, and postoperative histopathological work-up of the resected specimen may contribute valuable information to this interesting discussion, suggesting that calcaneal SBC and IOL may share a common pathogenesis, and that (calcaneal) IOL may develop from SBC.

## 2. Materials and Methods

Between 2012 and 2019, a total of 25 benign osteolytic bone tumors consisting of 17 SBCs and 8 IOLs were treated by A.T. with endoscopic resection and grafting. All cases of SBC or IOL were confirmed histopathologically. We excluded all cases of calcaneal ganglion cysts and other (pseudo-) tumorous lesions which are commonly seen in Diard´s Area 6 and must be distinguished from calcaneal SBC and IOL [37]. Overall, there were 17 male and 8 female patients with a mean age of 24.4y (range 12–62). There were 12 right and 13 left feet involved, and there were no bilateral cases. Patients with SBC (*n* = 17) had a mean age of 17.0y (range 12–41), with 12 male and 6 female patients being affected. Patients with IOLs (*n* = 8) had a mean age of 38.0 years old at the time of surgery (range 19–62), and there were 6 male and 2 female patients in this subgroup (Table 1).

Thirteen out of the 25 cases were incidental findings (52%). In 7 of the 13 cases, an MRI was performed after an ankle sprain. The remaining 6 MRIs were performed for the following reasons: achillodynia (2×), after a medial malleolar fracture, insect bite, tarsal coalition, and after whole-body computed tomography in a polytrauma patient (Table 1). For all cases, a preoperative MRI was performed in addition to plain radiographs. Due to their characteristic appearances on MRI, bioptical verification of the diagnosis was not required in any case. The analysis of the available imaging was performed by a musculoskeletal tumor radiologist with specialized training and the orthopedic tumor surgeon (AT). All cases were repeatedly discussed in a multi-disciplinary tumor board.

Surgery was indicated for the following reasons: pain (*n* = 10), tumor size with increased risk of a pathological fracture (*n* = 10), or at the explicit request of the patient in the case of tumor anxiety (*n* = 5). In cases of heel pain, all relevant differential diagnoses had been excluded through repeated clinical assessment and imaging diagnostics. The critical size for an elevated risk of fracture was defined as 100% of the intracalcaneal cross-section in the coronary plane and at least 30% in the sagittal plane of the lesion, according to the definition of Pogoda [17]. If the size of the lytic lesion did not warrant a recommendation for prophylactic stabilization, the affected patient was offered clinical and radiologic follow-up. As mentioned before, 5 patients with a relatively small tumor size opted for endoscopic tumor resection due to tumor anxiety after careful consideration of the available findings. Alternatively, bioptic verification of the diagnosis and follow-up controls were not requested.

After a detailed explanation of the possible advantages and disadvantages of the respective filling materials, the affected patient (in the case of minors, their parents) could decide whether to use allogenic bone or injectable bone substitute. Our hypothesis was that the use of injectable bone substitute might decrease the surgery duration and allow for early full weight-bearing. Patients who requested early full weight-bearing were recommended to have IBS as the filling material. Moreover, some patients preferred to have artificial bone substitute over allogenic bone for personal reasons and therefore opted for IBS as a filling material. ACB was recommended for all other patients based on our previous experience regarding osseous integration and the current state of the literature on calcaneal SBC, which suggests that cancellous bone is beneficial in terms of recurrence [12].

Autologous bone grafting was also discussed but not recommended due to the associated donor site morbidity. No randomization was performed.

A total of 12 patients with a mean age of 17.9 years (range 12–34) received allogenic cancellous bone (ACB, group A), and 13 patients with a mean age of 30.4 years (range 12–62) received injectable bone substitute (IBS, group B). In Table 1, patients who received allogenic bone appear on white background, and cases with injectable bone substitute are grayed out.

For grafting, allogenic bone in the form of cancellous bone was used for plombage of the cavity in 10 cases (DIZG, Berlin, Germany and ReadiGraft^®^ Cancellous Chips, LifeNet Health, Vienne, Austria), and injectable demineralized bone matrix was used in 2 cases (DIZG, Berlin, Germany). Four different types of injectable bone substitute were used: Cerament© (hydroxyapatite and calcium sulfate, Bonesupport, Lund, Sweden) in 10 cases, Pro-Dense© (calcium sulfate and calcium phosphate, Wright Medical, Memphis, TN, USA) in 1 case, QuickSet© calcium phosphate bone cement, Arthrex, Naples, FL, USA) in 1 case, and Innotere© (calcium phosphate bone cement, Arthrex, Naples, FL, USA) in 1 case.

The number of different bone graft substitutes can be explained by the availability and surgeons’ expectations regarding the osseous integration of the filling material. The first two cases using Pro-Dense and Quick-Set showed good applicability and handling but poor osteointegration over the initial course of time. The use of Cerament was expected to improve osseous integration. Due to its poor results with high complication rates, the use of this filling material was abandoned, and another IBS (Innotere) was used for the last case upon the patient’s request.

Depending on the product used, curing of the bone substitute differs slightly:

For Cerament©, the setting time is specified by the manufacturer as 15 min, and the initial compressive strength is 10–75 MPa (wet conditions–dry conditions). For Innotere©, a setting time of 24 h is given until the compression strength of cancellous bone is reached (10 MPa). For ProDense, the manufacturer specifies a compressive strength of 40 MPa after 2 h in wet conditions. Quickset is reported to reach a compression strength of 24 MPA at 24 h after implantation. After using injectable bone substitute, limited weight-bearing (15 kg) was recommended until wound healing was completed (2 weeks). When using allogenic bone for grafting, 6 weeks of limited weight bearing was indicated.

A radiologic follow-up was routinely performed 6 weeks, 12 weeks, and 12 months postop with plain radiography. To rule out recurrence, MRI imaging was indicated for all cases demonstrating a healing result with a defect on conventional radiographs (types Neer B–D).

The classification of the osseous healing outcome was based on the modified Neer classification (Table 2). The modified Neer classification has previously been used for of any osseous localization [51,52,53]. Type A describes a healed cyst filled with new bone, allowing a small radiolucent area of less than 1cm. With our modification of the classification, we only accepted complete osseus healing as type A (without any radiolucent area). Calcaneal SBC is sometimes only 3–4 cm in size. Therefore, we excluded any defect healing with a radiolucent area <1 cm for the type A classification, as this would correspond to a type B lesion in small SBCs.

Pre- and postoperative imaging with plain X-rays and MRI was retrospectively analyzed to assess tumor size, osseous consolidation, and tumor recurrence. A retrospective chart analysis focusing on adverse intra- and perioperative events and other complications associated to the surgical procedure was performed using the modified Clavien–Dindo classification [54,55,56]. Complications were defined as any deviation from the normal postoperative course [54]. Disturbed wound healing (DWH) was defined according to the definition of Dirschinger et al. for elective foot surgery [57]. Approval for the study was obtained from our institutional review board.

The surgery duration was recorded in minutes. All but one ossoscopy was performed as a standalone procedure. In one case, endoscopic tumor resection was performed in combination with a joint preserving surgery for tarsal coalition. For this case, no isolated surgical time was available for the endoscopic procedure.

### 2.1. Surgical Technique

**Figure 2:**
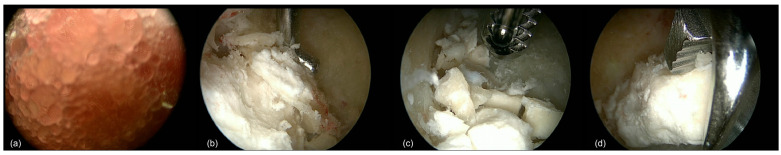
After the introduction of the scope into the bone cavity, vision was often impaired by fat tissue in the case of IOL (**a**). After thorough irrigation and endoscopic removal of the fat tissue using an arthroscopic shaver, residual calcifications were removed using an arthroscopic grasper or shaver (**b**–**d**).

**Figure 3:**
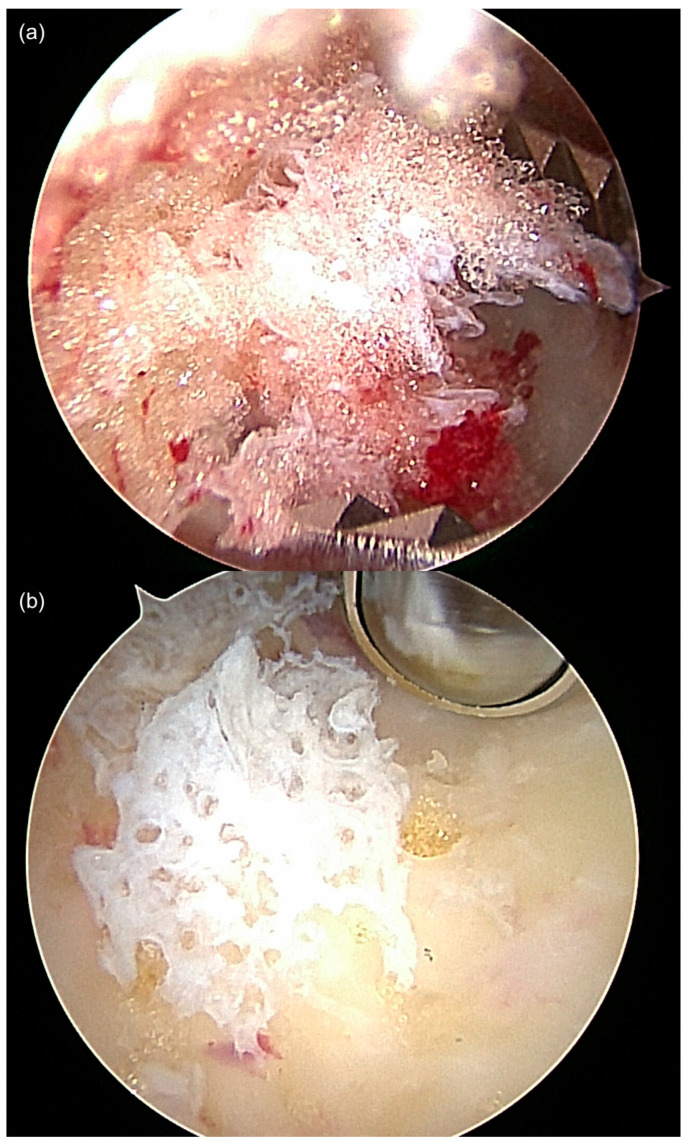
Often, typical features of IOL ((**a**), fat tissue) and SBC ((**b**) membrane/cyst lining) were both observed in parallel in calcaneal ossocopy, suggesting a common pathogenesis and transitional forms of SBC and IOL. Both images are from the same ossoscopy (case 12).

The surgical technique has been previously described by the authors [15,24,58]. The type of anesthetic procedure was determined by the patient and the anesthesiologist. The patient was placed in a stable lateral position on a radiolucent table. The dimensions of the bone lesion were marked on the skin of the lateral rear foot with a sterile pen under fluoroscopic control. The two portals for ossoscopy were marked according to the size of the cystic bone lesion. A tourniquet was used to reduce bleeding into the bone cavity and to facilitate visualization. After skin incision and blunt dissection of the underlying soft tissue, the thinned-out cortex was penetrated with a semi-sharp obturator before introducing the sheath for a 2.7 or 4 mm scope into the cavity. During blunt dissection to the lateral wall of the calcaneal bone, care was taken not to harm the sural nerve and the peroneal tendons. Contrary to ossoscopy of the calcaneal bone for a simple bone cyst, clear vision of the bone cavity in IOL can only be achieved after a second portal has been established and thorough endoscopic irrigation has been performed. Therefore, loose lipomatous tissue was washed out, and the typical calcified areas of intraosseous lipoma became visible. The calcifications were cleaned out using an arthroscopic shaver, and larger pieces were grasped using an arthroscopic punch or grasper (Figure 2). Often, a tennisnet-like pseudo-membrane covered the walls of the cavity. This membrane is common for SBC but can also be present in calcaneal lipoma, suggesting their common etiology (Figure 3). The membrane was completely resected to prevent tumor recurrence. Any visible tumor tissue was completely removed and sent for histopathological analysis. Injection of radiopaque contrast medium verified the integrity of the bone cavity (no accidental damage and leakage) and helped determine the amount of injectable bone substitute needed to completely fill the lesion.

**Figure 4:**
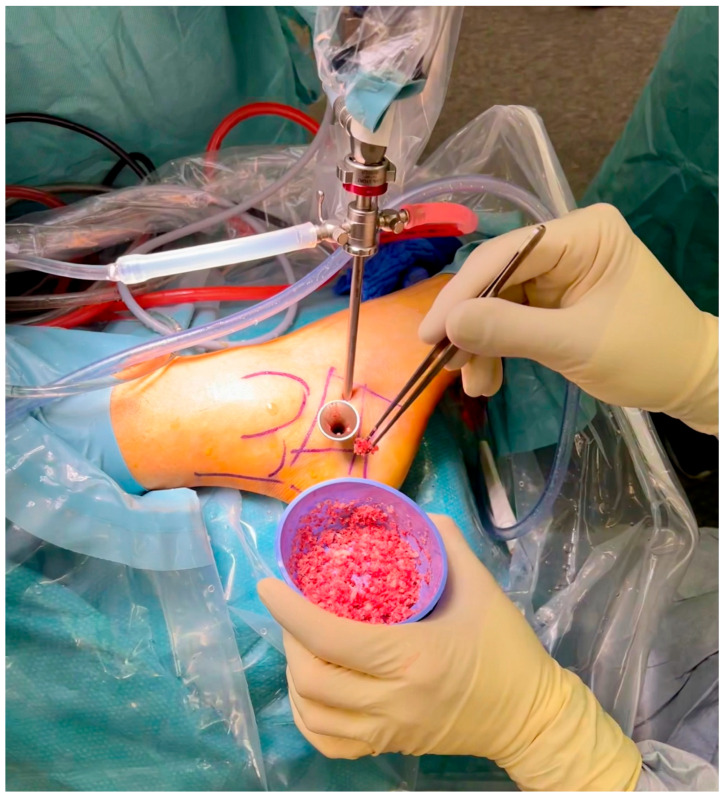
A *Hartmann* ear septum borrowed from ENT surgery facilitated the insertion of the allogeneic cancellous bone into the bone cavity.

**Figure 5:**
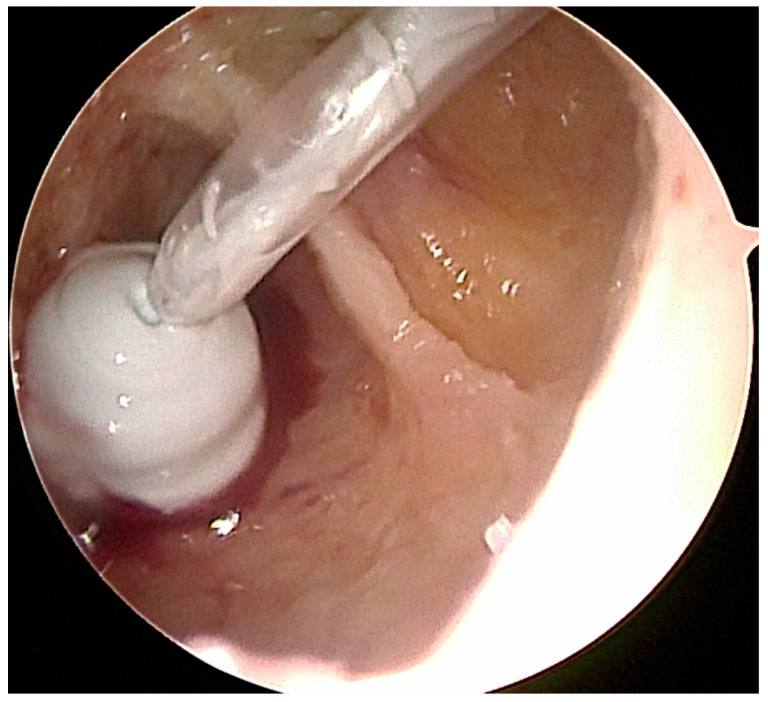
Injection of the resorbable bone substitute (in this case Cerament©) was visualized directly under endoscopic (and fluoroscopic) control.

Before grafting with allogenic bone or injectable bone substitute, the cavity was rinsed with 95% ethanol as a local adjuvant therapy, ensuring denaturation of the remaining microscopic cyst membranes and tumor tissue. The application time for the ethanol did not exceed 1–2 min, and after intermittent thorough irrigation with sterile saline solution, this procedure was repeated 2–3 times [52,59,60,61]. The ethanol did not come into contact with any soft tissue to avoid damage to sensible structures such as the sural nerve. For easy application of the allogenic cancellous bone chips, a small 4 mm *Hartmann* ear speculum (Aesculap, Tuttlingen, Germany) was introduced through one of the ossoscopy portals (Figure 4). In our experience, this device has proven to be easier to handle compared to previously used instruments such as a pedicle filler (borrowed from spine surgery) or a hollow bone punch originally used for biopsies. Under endoscopic vision through the second portal, impaction of the bone graft was performed intermittently. Alternatively, injectable bone substitute was applied under direct endoscopic and fluroroscopic control (Figure 5). Finally, both portals were sealed with a collagen sponge to avoid accidental leakage of the graft material. After wound closure, the foot was immobilized in a semi-rigid lower leg orthosis. Partial weight bearing was advised for 6 weeks for all cases treated with allogenic bone and for two weeks for those treated with injectable bone substitute (until the removal of the skin sutures).

### 2.2. Statistics

The parameters were expressed as mean, standard deviation (SD) and range (min./max.). A Fisher´s exact test was used to determine the statistical significance of the differences between Group A and Group B, as well as between the different Neer types of osseous healing. A *p*-value < 0.05 was used to define statistical significance. The statistics were performed by an independent statistician using Microsoft Excel© and RStudio software.

## 3. Results

**Figure 6:**
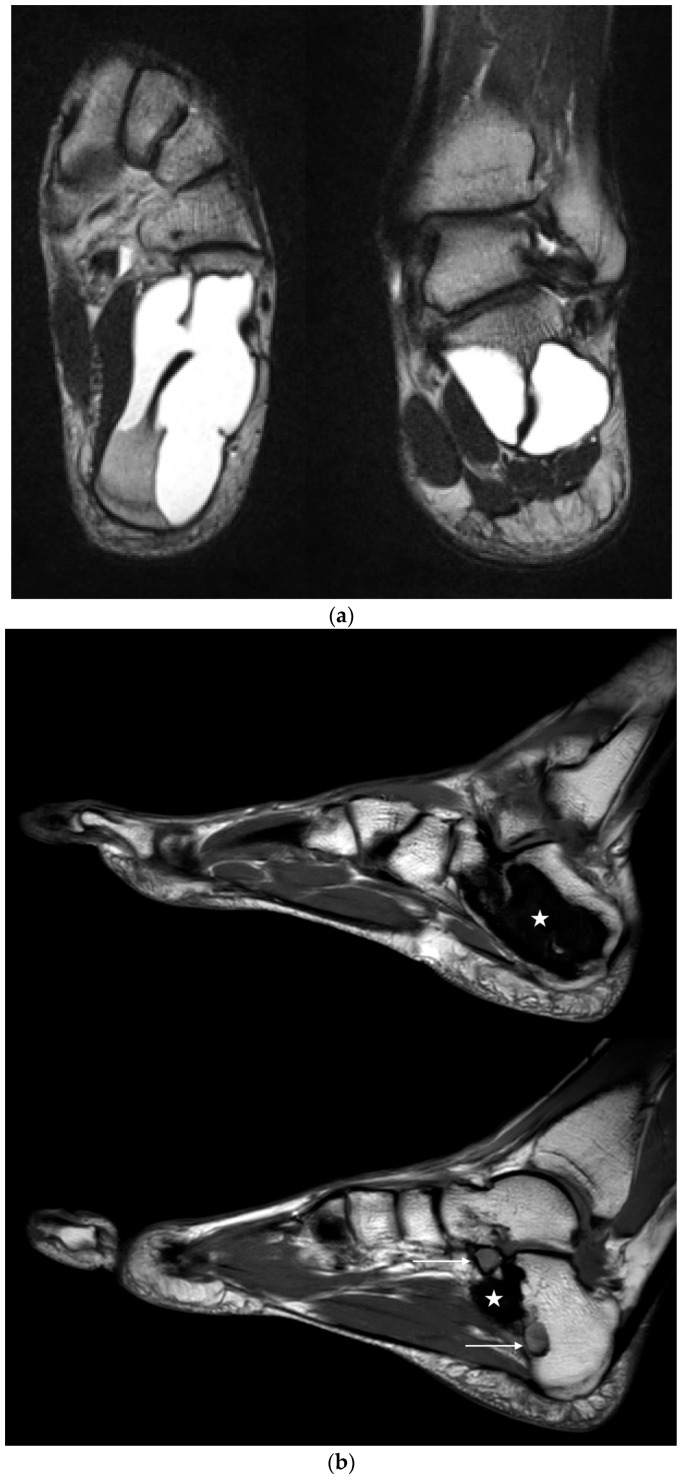
(**a**) Axial and coronal T2 TSE-weighted MRI showing a large SBC in a 31-year-old professional ballet dancer (case 6). After endoscopic resection, the bone cavity was filled with injectable bone substitute. (**b**) Sagittal T1-weighted MRI performed 91 months postop, showing two small areas of recurrence of SBC at the medial aspect (bottom image, arrows). The large cyst cavity was filled with QuickSet© injectable bone substitute (marked with a star), showing no signs of osseous remodeling more than 7.5 years after implantation. The patient is currently free of symptoms and still performs at the highest level of professional ballet dancing.

The mean follow-up (f/u) of 24/25 patients was 24.5 months (range 12–91). One patient (case 21) was lost to f/u after the first control six weeks postop. Patients showing complete osseous healing without any defect (Neer type A) in conventional X-rays and no symptoms one year postop were not required to undergo further imaging controls by our clinic, but were contacted by phone and/or mail in January 2021 asking for any additional imaging that was performed in the meantime. A return postage-paid envelope was mailed to the patients requesting the additional imaging. In two cases, the imaging was transmitted digitally. As a result, five more MRI follow-ups of were acquired for the initial Neer type A cases. Only one case (case 6) with MRI performed 91 months after index surgery showed small areas of recurrent SBC (Figure 6a,b), corresponding to a Neer type B healing. In the four remaining cases of initial type A healing, MRI with a mean f/u of 51.2 months (range 36–60) confirmed a complete healing.

### 3.1. Osseous Healing and Amount of Filling Material

Table 3 demonstrates the osseous healing results classified according to the modified Neer classification and the corresponding follow-up intervals at which the final results were obtained. Overall, 16 cases showed type A healing, four cases showed type B healing, one case showed type C healing, and five cases showed type C healing outcomes.

Moreover, where available, the amount of filling material used is noted (Table 3). For injectable bone substitute, the exact amount injected was taken from the surgical report. In cases involving allogenic bone, impaction grafting was routinely performed to enhance osseous healing [12]. In group A, the correct volume of the bone cavity could only be assessed based on the amount of contrast medium injected into the bone void prior to definite filling. This step of the procedure was routinely performed to rule out any iatrogenic damage and unwanted leakage (see Section 2.1). In 6/25 cases, no information on the amount of the filling material was available retrospectively (n/a).

Statistically significant differences were observed in the healing outcome between type Neer A and higher Neer types (B, C, or D) in favor of the use of allogeneic cancellous bone versus injectable bone substitute (Fisher’s test, *p* = 0.011).

### 3.2. Complications

Table 4 gives an overview of the modified Clavien–Dindo classification used for this study. Grades 4 and 5 of the original classification were not listed, as no such complications were observed (life-threatening complications or death) [54,55]. Table 3 lists the complications that occurred intraoperatively or postoperatively. Overall, there were 13 complications in 13 out of 25 patients (52%). There was one Stage 1 complication (CD1) involving delayed/disturbed wound healing (DWH, as defined by Dirschinger et al. [57]) without a change in postoperative care (case 16). There were ten Stage 2 complications (CD2). A total of 2/10 Stage 2 complications showed transient suralis neuropraxia, and two cases showed complications consisting of DWH with prolonged wound secretion attributed to the use of injectable DBM. The remaining six Stage 2 complications were “white-outs”, and exclusively observed in cases where Cerament© was used as the injectable bone substitute. This phenomenon has been previously described in the literature [62] and is well known to the manufacturer. In November 2018, Bonesupport issued a customer information stating that *“clinical experience has shown that in a few cases where the product is used for the treatment of aneurysmal bone cysts and other bone cysts that tend to produce large amounts of fluid, there is an increased risk of wound secretion, soft tissue inflammation, and wound healing problems when treated by open surgery“* [63]. We previously encountered this complication for different tumor entities (e.g., chondrosarcoma) and indications (e.g., bone defects in trauma cases or neuro-osteoarthropathy). Since the containment of the bone cavity is minimally affected by an endoscopic procedure, we did not expect to encounter this problem in calcaneal ossoscopy. Repeatedly, (superficial) wound infection was initially suspected by either the attending surgeon or family doctor, leading to additional clinic visits, microbiological swab tests, and/or oral antibiotic therapy.

**Figure 7:**
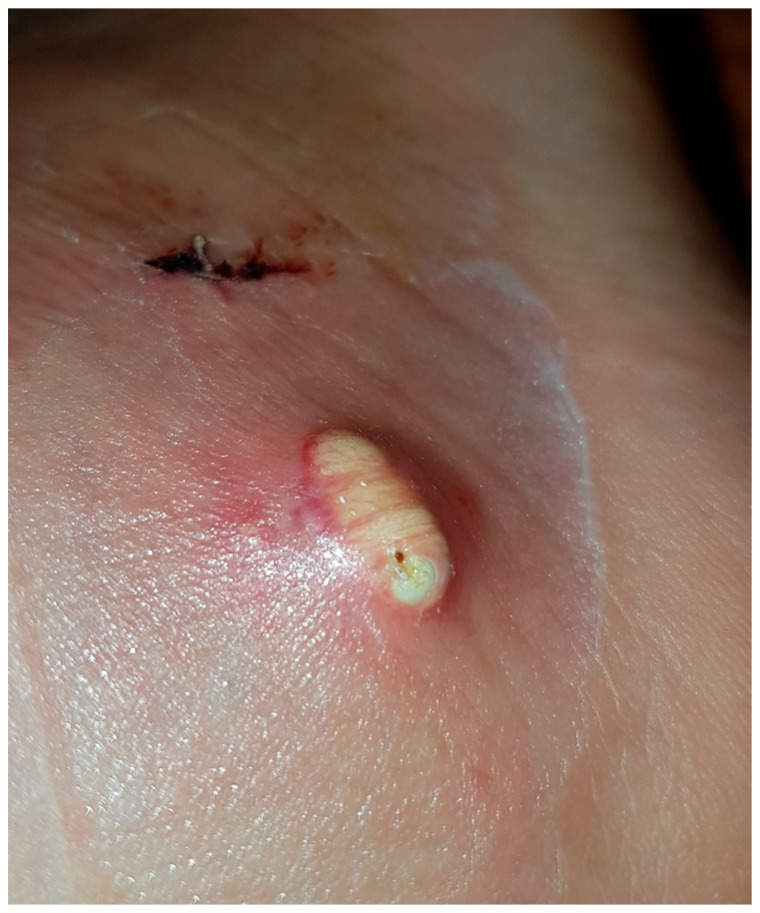
Case 24: Persistent wound drainage resembling pus led to revision surgery 14 days after index surgery for suspected deep infection. Intraoperative microbiological testing did not prove infection. “White-out” after injection of Cerament© was identified as the cause of the complication.

There were two Stage 3 complications (CD3), both requiring revision surgery. The first case was caused by iatrogenic damage of the medial calcaneal cortex during endoscopic tumor resection and undetected leakage of the injectable bone substitute (case 6). Due to inadequate pain and swelling on the medial aspect of the hindfoot, CT imaging was performed and revision surgery was necessary to remove the leaked material on the medial side two days after the index surgery. The second Stage 3 complication required open surgical revision surgery (performed elsewhere) for suspected deep infection and severe inflammation of the surrounding soft tissue (case 24, Figure 7). Microbiological testing showed no evidence of bacterial growth, suggesting that the findings were also associated with a “white-out” phenomenon.

In group A (ACB, cases on white background in Table 2), there were 2/12 complications (one DWH and one suralis neuropraxia). In group B (IBS, grayed out cases in Table 2), there were 11/13 (84.6%) patients with complications, predominantly classified as CD2 and CD3 complications (10/13). Regarding complications, there was a statistically significant difference between the two groups (*p* = 0.0012).

The contingency table was used to show the absolute frequency of use of ACB and IBS, as well as complications between the two groups (Table 5). Fisher’s exact tests were used to calculate the *p*-values between the two groups (due to small sample sizes).

### 3.3. Surgery Duration

Overall, the mean operation time was 75 min (range 3–133) and 72 min (range 35–103) for group A (ACB), and 84 min (range 45–133) for group B (IBS), showing no statistically significant difference (*p* < 0.05). For group B, the surgery duration for the isolated ossoscopy was not available retrospectively in two instances (cases 16 and 21). One of those procedures (case 21) was performed in combination with a joint-preserving PCFD correction.

### 3.4. Histological Findings

Interestingly, two cases of IOL (cases 5 and 12) with characteristic preoperative MRI findings were histopathologically diagnosed as SBC. One additional case of MRI-diagnosed SBC showed membranous material with a histiocyte-resorptive reaction, consistent with SBC (case 11). Another case had material from a hemorrhaged lesion along with several bony particles (case 8), but no clear signs of lipoma of the bone. The absence of lipomatous tissue can be explained by the surgical technique. During endoscopic resection of IOL, the lipomatous tissue from the bone cavity is often washed out until a clear ossoscopic view is established and appropriate specimens are obtained (see Section 2.1). This problem was addressed later in the study (starting with case 13) by taking biopsies blindly once access to the tumorous lesion was established and prior to introduction of the scope. Our findings support the observation that SBCs can heal with fatty conversion of the cystic cavity, resulting in partly cystic remnants that may eventually transform to IOL (Figure 3). We agree with the findings of Tins at al. that at least part of the so-called IOLs are in fact, healed simple bone cysts [50].

## 4. Discussion

Are simple bone cyst and intraosseous lipoma two different entities?

**Figure 8:**
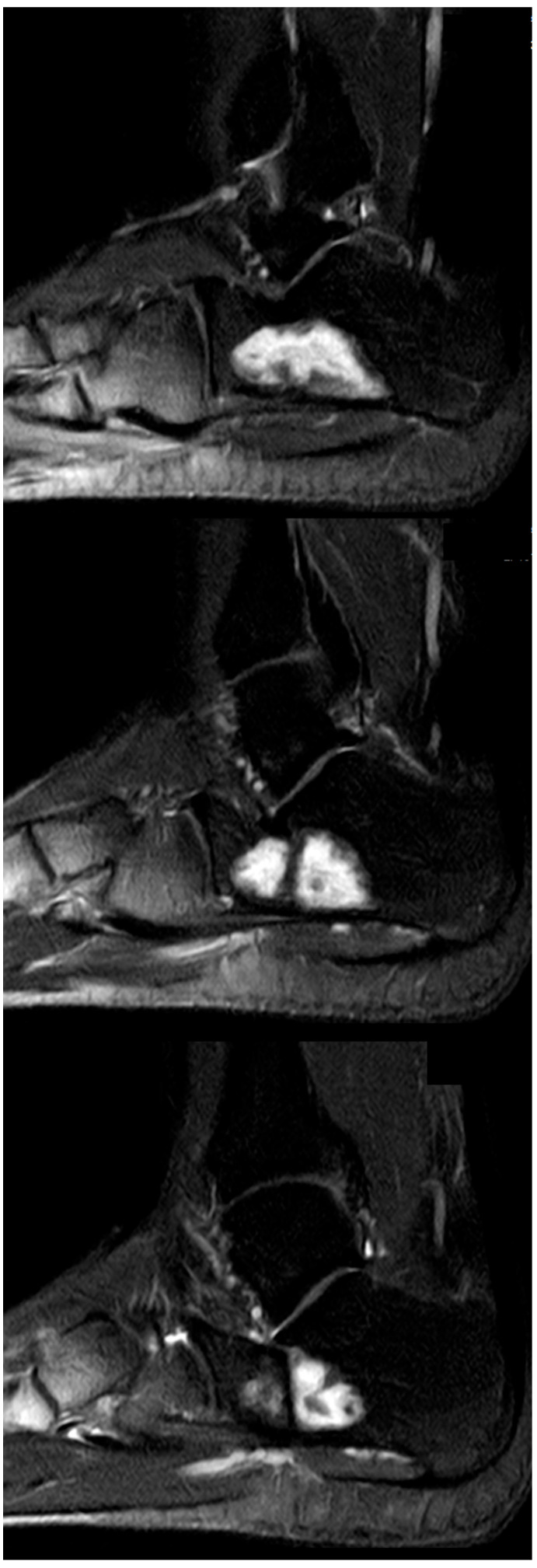
Preoperative MRI of case 12 (19-year-old male patient) showing a calcaneal IOL with cystic changes corresponding to a Milgram Stage 3 type [29]. However, histopathological examination of the tissue samples taken from the bone cavity revealed SBC.

Preoperative imaging with plain radiographs and MRI led to the diagnosis of 17 SBCs and eight IOLs. Both entities exhibited a characteristic, almost pathognomonic appearance on MRI, making bioptic verification of the diagnosis unnecessary. Often times, MRI revealed a mixed form of SBC and IOL, showing lipomatous tissue enclosing central cystic areas (Figure 8). This has been commonly described as the evolution of an IOL (Milgram Stage 3 [18]) or involution from other preexisting bone lesions [28,36,46,47], suggesting that IOL develops from precursor lesions. It should be noted that in the current 2020 edition of the WHO classification of soft tissue and bone tumors, SBC and IOL are listed as separate entities and their etiology is considered unknown [1]. However, several publications have documented the transition of SBC to IOL over time, suggesting that IOL may actually represent a developmental form of SBC [10,30,50,64].

While MRI allows differentiation between cystic from lipomatous areas of the lesion, histopathological analysis is required to verify the correct diagnosis. Direct endoscopic visualization of the lesion can help to correctly assess mixed and transitional forms of SBC and IOL macroscopically (Figure 3). The high resolution of modern scopes (4K) and the magnification factor provide a much more detailed view of the findings during ossoscopy compared to open surgical resection. Thus, the tennis net-like membrane of SBC and other components of the lesion can clearly be identified.

Two cases initially diagnosed as IOLs based on MRI turned out to be SBC in the histopathological analysis (case 5 and 12). Another case of suspected IOL demonstrated membranous material with a histiocyte-resorptive reaction but no cholesterol clefts (which are typical for SBC) upon histopathological evaluation, resembling SBC (case 10).

Both our endoscopic and histopathological results support the theory proposed by other authors such as Malghem, Tins, and Kawaguchi that (calcaneal) IOL develops form SBC [10,30,50,64].

Which option should be used now: allogenic cancellous bone or injectable bone substitute?

A systematic review on the treatment of unicameral bone cysts of the calcaneus conducted by Levy et al. from 2015 concluded that open curettage with autograft bone augmentation is the most effective procedure. Unsurprisingly, autograft procedures resulted in significantly greater radiographic healing compared with allografting. The review included only one publication on endoscopic curettage and calcium–phosphate injection, and two more reports with two cases each treated with open curettage and calcium phosphate or calcium sulfate, respectively [12].

In a recent study by Ma et al., *45S5 Bioglass* was reported to better facilitate the formation of new bone with a faster drying time of the skin incision than allogenic bone in open curettage of 31 patients, including 18 cases of SBC and six cases of IOL with a f/u of 12 months [65]. Only plain radiographs were used to assess osseous integration of the filling material.

Karr et al. reported the use of Cerament© calcium sulfate/calcium phosphate bone void filler in a patient with bilateral calcaneal SBC, mixing the injectable bone substitute with ACB on one side, demonstrating complete trabecular regeneration at 2.3 years (right foot) and three years (left foot) postoperatively [66].

Aycan et al. reported on intralesional curettage and defect filling with cortico-spongeous allograft in 14 patients with calcaneal intraosseous lipoma. After a mean f/u of 84 months (range 18–108), no recurrence was observed [67]. Ulucay et al. reported on 21 cases of calcaneal IOL treated with cancellous iliac crest autografts after open curettage, and no recurrence was observed after a mean f/u of 94 months (range 45–143) [27].

Previously, we reported our findings of endoscopic resection and allografting for four cases of calcaneal IOL and six cases of SBC, all demonstrating complete osseous healing after a mean f/u of 19.8 months (range 9.5–39.3) [15].

In 2011, Yildirim performed a comparative study including 26 cases of calcaneal SBC. Thirteen feet underwent open surgical curettage, and 13 cases underwent endoscopic curettage. Both groups received allografting. The operating time and mean length of stay were significantly shorter in the endoscopic group. The time to healing was similar in both groups. The overall success rate was higher for the endoscopic group (100%, 13 of 13 vs. 92.3%, 12 of 13 cases), and there were no statistically significant differences regarding radiological healing [68].

Similarly, Nishimaura et al. published their findings on 16 calcaneal SBCs in 2016. Eight patients underwent an open procedure, and eight cases were treated with an endoscopic procedure. No significant difference between the two groups was observed in the operative time. The surgery duration for the endoscopic group was 56.1 ± 13.8 min, and no adverse effects were observed. However, the open group experienced one temporary irritation in the sural nerve area and one calcium phosphate cement leakage along the peroneal tendon sheath. The interval for a return to sports was significantly shorter in the endoscopic group. The authors concluded that endoscopic surgery is a useful approach for the treatment of calcaneal bone cysts, allowing early rehabilitation and an early return to sports without any adverse effects.

For better comparison, Table 6 lists all available studies on endoscopic resection of calcaneal SBC and/or IOL. To the best of our knowledge, our case series is the only study that routinely included MRI in follow-ups. As conventional radiography is insufficient for sufficiently assessing osseous integration of the graft or tumor recurrence, we strongly recommend including MRI in future studies.

A publication by Choi et al. on endoscopic tumor treatment of the skeleton was not included because the author failed to indicate the anatomic location of nine cases of SBC [80]. Surprisingly, no complications were reported in 14 publications that included a total of 61 cases of calcaneal SBC or IOL, with a mean f/u of 30.86 months (no recurrence, no DWH, no suralis neuropraxia). This discrepancy may be attributed to the type of imaging used in follow-ups (X-rays vs. MRI), the different definitions of osseous integration and recurrence, and the duration of the f/u.

Apart from the publication of Nishimura et al., only Farouk et al. provided information on their surgery duration for three cases of endoscopic resection of calcaneal IOL (45, 40, and 15 min, respectively). Grafting was performed in 2/3 cases with PMMA, while the third case did not receive any filling of the bone cavity. The mean surgery time in our series was 75.2 min. (range 35–133) and 71.7 min (range 35–103) for group A (ACB), and 84.2 min (range 45–133) for group B (IBS). Our hypothesis that the use of injectable bone substitute would shorten the duration of the procedure compared with allogeneic bone grafting was not confirmed.

**Figure 9:**
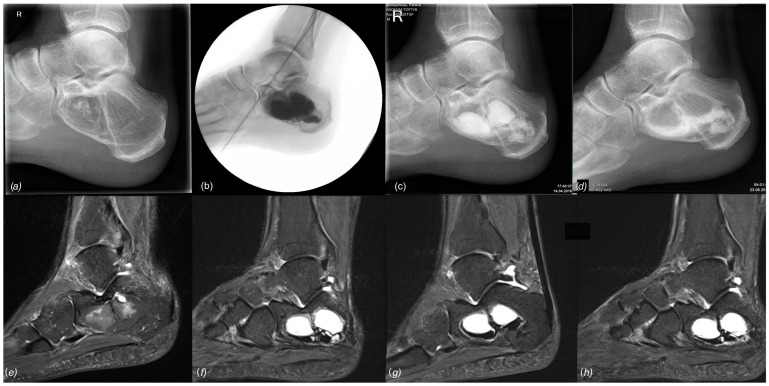
Top, left to right: (**a**) preoperative X-ray of a large IOL, Milgram Stage 3 (case 22); (**b**) intraoperative fluoroscopy, postoperative radiographs at 6 (**c**) and 12 (**d**) weeks postoperatively showing continued loss of the filling material, consistent with persistent wound drainage. Bottom, left to right: (**e**) preoperative MRI, (**f**) sagittal MRI 12 months postop, (**g**) 20 months postop, and (**h**) 32 months postop. All MRI are sagittal T1 TIRM weighted.

**Figure 10:**
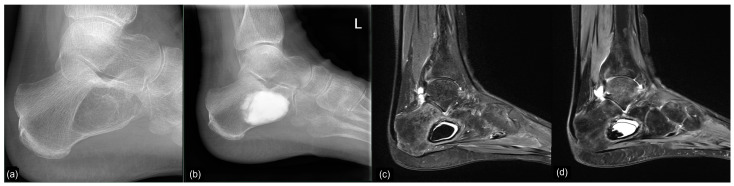
Conventional radiological and MR tomographic follow-up of case 23: (**a**) preop, (**b**) 3 days postop, (**c**) 8 weeks postop, and (**d**) 3.5 months postop. Leakage of Cerament© was observed clinically (“white-out”) and radiologically.

**Figure 11:**
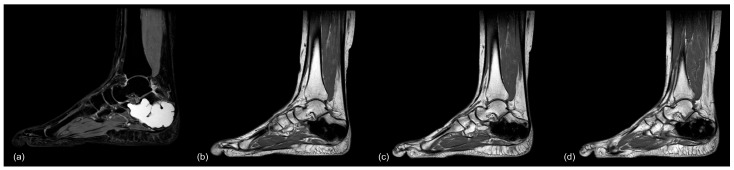
MR-imaging of case 26: (**a**) preop (PD TSE sag), (**b**) 6.5 months postop (T1 TSE sag), (**c**) 13 months postop (T1 TSE sag), and (**d**) 40 months postop (T1 TSE sag), showing no signs of osseous remodeling after more than 3 years postoperatively.

Complications were predominantly related to the use of Cerament©, leading to a 90% complication rate (9/10) in patients treated with this particular injectable bone substitute. These complications included DWH with “white-out” in 7/10 cases, one case of DWH without “white-out”, one revision surgery, and one suralis neuropraxia. As the “white-out” phenomenon has been repeatedly observed in various non-tumorous indications in our clinical practice, and excessive white drainage after the use of Cerament© does not seem to be limited to its use in aneurysmal bone cysts, we have stopped using this product entirely. Figure 9 and Figure 10 show examples of the gradual loss of Cerament© over time, leading to “white-out”, insufficient stabilization of the bone cavity, and cyst recurrence. These specific complications did not occur with other injectable bone substitutes. However, repeated MRI performed after treatment with two different types of calcium phosphate bone cement (case 6 and 25, Quickset© and Innotere©) did not demonstrate any postoperative signs of osseous ingrowth or bony transformation at 36 and 91 months (Figure 6b and Figure 11), respectively.

Aiba et al. proposed the use of curettage without additional grafting for simple bone cysts in various locations. They reported a recurrence rate of 18.9% (7/37 patients) when using endoscopic curettage as a stand-alone procedure. It is important to note that all recurrences occurred in tubular (*n* = 6) or flat bones (*n* = 1), and not in the calcaneus [53] (Table 6). Farouk et al. reported on one case of calcaneal SBC treated with endoscopic curettage without grafting [77]. Other reports of curettage without grafting exist for different locations, but are mostly limited to case reports [81,82]. Apart from the six cases of calcaneal SBC described in Aiba’s publication and one case published by Farouk et al., to our knowledge, there is no other literature supporting curettage without grafting at this site [53,77].

For large SBCs in the lower weight-bearing extremities, grafting is considered advantageous in regard to actual osseous healing. This hypothesis is supported by the findings of the only systematic review available on calcaneal SBC published by Levy et al. in 2015 [12]. Open curettage with bone augmentation demonstrated the best outcomes in their review. Nearly 80% of patients in the bone augmentation group experienced heel pain that had completely resolved after a mean duration of 3.3 ± 1.3 years. No clear distinction was found between autografting and allografting. No patients in either group experienced recurrence, complications, or reactions suggestive of graft rejection [12].

Intralesional tumor resection with thorough curettage of the simple bone cyst with complete removal of the inner cyst wall and cyst membrane is generally regarded as the key to preventing recurrence. The current literature on calcaneal SBC favors additional cancellous bone grafting over IBS or techniques without grafting (e.g., cannulated screw decompression or corticosteroid injections) [12,58].

### Strengths and Limitations

Although this is the largest case series on endoscopic resection of calcaneal SBC and IOL, identifying an exact difference between the two groups (statistical power) would require much larger sample sizes. This is true for most rare diseases and is unlikely to change in the foreseeable future. Calcaneal SBC proved to be the most common benign bone tumor in our publication on the distribution patterns of foot and ankle tumors (50 SBC out of 413 tumors overall) [6], but it was observed less frequently in Ruggieri’s analysis (51 SBC out of 1170 tumors) [7]. Nonetheless, it remains exceedingly rare outside of a university tumor center. A significant portion of SBC and IOL cases are diagnosed as incidental findings and do not require any form of therapy. This is why even a university musculoskeletal tumor center can only report a limited number of cases over a period of almost ten years. All surgeries were performed by the same surgeon (A.T.), ensuring a certain standard and reproducibility of the procedure and eliminating heterogeneous treatment algorithms that could possibly influence outcome.

One limitation is the use of different types of injectable bone graft substitutes, preventing any general recommendation for or against their use in calcaneal ossoscopy. Nevertheless, IBS regularly showed prolonged wound drainage (Cerament©) or showed no signs of osseous integration after several years of f/u (ProDense©, Innotere©). The follow-up was inconsistent, both in terms of the type of imaging and the duration of follow-up. MRI was only routinely indicated if plain radiographs at 12 months post-op suggested incomplete healing. Still, 10/25 patients (40%) had (repeated) MRI with a mean f/u of 40.3 months (range 12–91) after surgery, providing a medium-term radiological outcome including MRI for a rare condition treated with an even rarer surgical procedure.

Most publications on calcaneal IOL consist of case reports with one or two cases. To the best of our knowledge, there are only three publications with a larger case load of calcaneal IOL, all of them reporting open surgical tumor resection [27,67,83]. Regarding endoscopic treatment of calcaneal IOL, this is the largest study to date.

Another limitation of this study is its retrospective, non-randomized design. Conducting a prospective, single-center study with sufficient statistical power is not feasible within a reasonable time frame for rare diseases such as calcaneal IOL or SBC. Patients were able to choose the respective filling material after careful consideration of possible advantages and disadvantages. Although both groups (ACB vs. IBS) had almost identical sample size, the inclusion of two different tumor entities could be criticized. Several publications and our own findings suggest that SBC undergoes a continuous lipomatous evolution over time, eventually transforming into IOL [10,30,50,64,84,85]. In fact, both tumors might be considered as different developmental stages of the same entity.

## 5. Conclusions

We conclude that endoscopic treatment of calcaneal SBC and IOL is a safe procedure with low complication rates when combined with the correct filling material. The use of Cerament© for calcaneal SBC and IOL is discouraged due to the high complication rates, particulatly the occurrence of “white-out” and cyst recurrence. Other injectable bone substitutes demonstrated no prolonged wound drainage but also no signs of osseous remodeling on repeated MRI examinations over the course of several years.

Over time, calcaneal SBC may transform into IOL while exhibiting specific radiologic, endoscopic, and histopathologic features of both entities next to each other.

## Figures and Tables

**Table 1:** Demographic information on all cases that received ossoscopy and were included in the study. Diagnoses provided using MRI (SBC or IOL), location, and incidental findings are listed. Patients receiving allogenic bone for grafting appear on a white background (group A), cases with injectable bone substitutes are grayed out (group B).

Case	Sex	Age of Patient	Follow-Up in Months	MRI Diagnosis	Location	Incidental Finding
1	m	14	12	SBC	r	yes, MRI after ankle sprain
2	f	15	12	SBC	l	no
3	m	12	23.7	SBC	l	no
4	m	18	12	SBC	r	yes, MRI after ankle sprain
5	m	28	12	IOL	l	no
6	f	31	91	SBC	l	no
7	f	34	12	IOL	r	yes, MRI after ankle sprain
8	m	16	23.7	SBC	l	no
9	f	15	12	SBC	r	yes, MRI after insect bite
10	m	26	12	IOL	r	yes, after medial malleolar fracture
11	m	15	12	SBC	l	yes, MRI after ankle sprain
12	m	19	12	IOL	r	no
13	m	20	60.4	SBC	l	yes, MRI after ankle sprain
14	m	12	54.2	SBC	r	yes, MRI for achillodynia
15	m	18	54.7	SBC	l	no
16	f	14	11.4	SBC	l	yes, MRI for tarsal coalition and PCFD
17	m	15	52.6	SBC	r	no
18	f	15	14.9	SBC	l	yes, MRI for achillodynia
19	m	21	12	SBC	l	yes, MRI after ankle sprain
20	m	14	12	SBC	r	no
21	m	52	12	IOL	r	no
22	m	62	20	IOL	r	yes, after whole body CT in a polytrauma patient
23	f	59	12	IOL	l	no
24	m	24	12	IOL	r	yes, MRI after ankle sprain
25	f	41	36	SBC	l	no

PCFD: Progressive Collapsing Foot Deformity. r: Right. l: left.

**Table 2:** Modified Neer classification. Contrary to previous reports on a modification of the original Neer classification, only complete osseous healing qualified as Type A healing in our case series. Amongst others, the authors Hou and Aiba previously described type A as a healed cyst filled with new bone, allowing a small radiolucent area of less than 1 cm [52,53].

Classification	Description	Details
A	Healed	Cyst filled completely with new bone
B	Healed with a defect	Radiolucent area (<50% diameter) with enough cortical thickness
C	Persistent cyst	Radiolucent area (≥50% diameter) with thin cortical rim
D	Recurrent cyst	Cyst reaapears in the obliterated area or increased residual radiolucent area

**Table 3:** Table 3 provides information on the surgery duration, the type and amount of filling material, the type and time of radiologic follow-up, the healing result according to the modified Neer classification, and any surgery-associated complications staged with a modified Clavien–Dindo classification for each case. (ACB = allogenic cancellous bone, DBM = demineralized bone matrix, DWH = disturbed wound healing).

Case	Sex	Age of Patient	Surgery Duration in min.	Filling Material and Amount Used in ccm /mL	Radiological f/u (in m), Type of Imaging	Healing Result	Complication and Clavien-Dindo Grade (CD)
1	m	14	102	ACB, (n/a)	12, X-ray	Neer A	none
2	f	15	79	ACB, 30 ccm	12, X-ray	Neer A	none
3	m	12	47	ACB, (n/a)	24, X-ray	Neer A	none
4	m	18	35	ACB, (n/a)	12, X-ray	Neer A	none
5	m	28	66	ProDense, (n/a)	13, X-ray	Neer A	DWH with prolonged wound secretion, CD2
6	f	31	133	QuickSet, 42 mL	91, MRI	Neer B	Leakage of injectable bone substitute medially, surgical revision, CD3
7	f	34	89	DBM, 30 ccm	12, X-ray	Neer A	none
8	m	16	70	ACB, (n/a)	24, X-ray	Neer A	transient sural nerve neuropraxia, CD2
9	f	15	65	DBM, 30 ccm	12, X-ray	Neer B	DWH with prolonged wound secretion, CD2
10	m	26	103	ACB, 30 ccm	12, X-ray	Neer A	none
11	m	15	59	ACB, (n/a)	12, X-ray	Neer A	none
12	m	19	103	Cermanet, 8 mL	12, X-ray	Neer B	DWH with “white-out”, CD2
13	m	20	52	Cermanet, 5 mL	60, MRI	Neer A	DWH with “white-out”, CD2
14	m	12	45	Cermanet, 4 mL	54, MRI	Neer A	DWH with “white-out”, CD2
15	m	18	68	Cermanet, 12 mL	54, MRI	Neer A	transient sural nerve neuropraxia, CD2
16	f	14	n/a	Cermanet, 4 mL	11, MRI	Neer B	DWH, CD1
17	m	15	47	Cermanet, 6 mL	53, MRI	Neer C	none
18	f	15	66	ACB, 6 mL	15, MRI	Neer A	none
19	m	21	76	ACB 12 mL	13, X-ray	Neer A	none
20	m	14	70	ACB, 30 ccm	12, X-ray	Neer A	none
21	m	52	n/a	Cerament, 5 mL	lost to f/u, X-ray	Neer D	DWH with “white-out”, CD2
22	m	62	133	Cerament, 22 mL	20, MRI	Neer D	DWH with “white-out”, CD2
23	f	59	96	Cerament, 15 mL	12, MRI	Neer D	DWH with “white-out”, CD2
24	m	24	91	Cerament, 12 mL	12, MRI	Neer D	“white-out” with suspected surgical wound infection, CD3, 1× revision surgery
25	f	41	92	Innotere, 32 mL	36, MRI	Neer A	none

**Table 4:** Modified Clavien–Dindo classification of surgery-related complications after calcaneal ossoscopy.

Grade	Definition	Specific Complication
1	A complication that requires no treatment and has no clinical relevance; there is no deviation from routine follow-up during the postoperative period;	wound problems (DWH) not requiring a change in postoperative care
2	A deviation from the normal postoperative course (including unplanned clinic visits) that requires outpatient treatment: either pharmacological or close monitoring as an outpatient	Superficial wound infection (additional clinic visits), transient neurapraxia, “white-out“
3	A complication that is treatable but requires surgical, or radiographic procedure(s), or an unplanned hospital readmission	deep infection, leakage from injectable bone susbtitute, tumor recurrence

**Table 5:** A contingency table was used to show the absolute frequency of use of ACB and IBS, as well as complications between the two groups.

	Complications, *n*	No Complications, *n*	Total
ACB, *n*	2	10	12
IBS, *n*	11	2	13
total	13	12	

ACB = allogenic cancellous bone, IBS = injectable bone substitute. Fisher’s exact test: two-tailed *p*-value equals 0.0012. The association between rows (groups) and columns (outcomes) is considered to be very statistically significant.

**Table 6:** Literature overview of publications reporting on cases of calcaneal endoscopy for SBC or/and IOL, including the number of cases treated, filling material, follow-up, and complications encountered.

Author, Year, Journal	Number of Cases Included (SBC/IOL)	Filling Material	Healing Rate in %, f/u in Months	Complications
Bonnel, 1999, Acta Orthop Belgica [69]	1 SBC	ACB + autologous iliac crest bone marrow aspirate	100%, 20 m	none
Mainard, 2006, JFAS [70]	1 SBC	calcium phosphate cement, Eurobone©	100%, 12 m	none
Alvarez, 2007, FAI [71]	1 SBC	ACB + autologous iliac crest bone marrow aspirate	n/a	n/a
Roth, 2010, J Orthop Scie [72]	2 SBC	ACB	100%, ø f/u 35 m	none
Innami, 2011, Am J Sports Med [73]	10 SBC	calcium phosphate cement, Biopex©	100%, ø /f/u 36,2	none
Yildirim, 2010, JFAS [74]	5 SBC	ACB	100%, 12 m	none
Yildirim, 2011, JBJS Br [68]	13 SBC *	ACB	100%, ø f/u 28.7 m	none
Nishimura, 2016, JFAS [75]	6 SBC *	calcium phosphate cement, Biopex©	100%, ø f/u 29.3 m	none
Toepfer, 2016, Springer Plus [15]	10 (6 SBC, 4 IOL)	ACB	100%, ø f/u 19.8 m	none
Stoica, 2017, Rom J Morphol Embryol [76]	1 SBC	ACB	100%, 60 m	none
Aiba, 2018, JOSR [53]	6 SBC *	No filling, only curettage	100%, ø f/u 33.8 m	none
Farouk, 2018 SICOT J [77]	3 IOL *	2× PMMA bone cement, 1× no filling	100% ø f/u 42.5 m, 1 case lost to f/u	none
Megremis, 2021, Tech Foot and Ankle [78]	1 SBC	autologous and allogenic cancellous bone, DBM	100%, 60 m	none
Yi, 2021, medicina [79]	1 SBC	ACB	100% 12 m	none

Cases marked with an asterisk (*) represent publications including subgroups of endoscopic resection of calcaneal SBC or IOL. ACB: allogenic cancellous bone. DBM: demineralized bone matrix. PMMA: polymethyl methacrylate.

## Data Availability

All data generated or analyzed during this study are included in this published article. The datasets used and/or analyzed during the current study are also available from the corresponding author upon reasonable request.

## Abbreviations

ACB	allogenic cancellous bone
CD	Clavien–Dindo (classification)
DBM	demineralized bone matrix
DWH	disturbed wound healing
f/u	follow-up
IBS	injectable bone substitute
IOL	intra-osseous lipoma
MRI	magnetic resonance imaging
PCFD	progressive collapsing foot deformity
SBC	simple bone cyst
TIRM	Turbo inversion recovery magnitude
WHO	World Health Organization

## References

[1] Reith J., Bloem J., Forsyth R. (2020). WHO Classification of Tumours Soft Tissue and Bone Tumours.

[2] Mirra J. (1989). Bone Tumors: Clinical, Rardiologic, and Pathologic Correlations.

[3] Diard F., Hauger O., Moinard M., Brunot S., Marcet B. (2007). Pseudo-cysts, lipomas, infarcts and simple cysts of the calcaneus: Are there different or related lesions?. JBR-BTR Organe Soc. R. Belg. Radiol..

[4] Moreau G., Letts M. (1994). Unicameral bone cyst of the calcaneus in children. J. Pediatr. Orthop..

[5] Kadhim M., Thacker M., Kadhim A., Holmes L. (2014). Treatment of unicameral bone cyst: Systematic review and meta analysis. J. Child. Orthop..

[6] Toepfer A., Harrasser N., Recker M., Lenze U., Pohlig F., Gerdesmeyer L., von Eisenhart-Rothe R. (2018). Distribution patterns of foot and ankle tumors: A university tumor institute experience. BMC Cancer.

[7] Ruggieri P., Angelini A., Jorge F.D., Maraldi M., Giannini S. (2014). Review of foot tumors seen in a University tumor Institute. J. Foot Ankle Surg..

[8] Pretell-Mazzini J., Murphy R.F., Kushare I., Dormans J.P. (2014). Unicameral bone cysts: General characteristics and management controversies. J. Am. Acad. Orthop. Surg..

[9] Takada J., Hoshi M., Oebisu N., Ieguchi M., Kakehashi A., Wanibuchi H., Nakamura H. (2014). A comparative study of clinicopathological features between simple bone cysts of the calcaneus and the long bone. Foot Ankle Int..

[10] Malghem J., Lecouvet F., Vande Berg B. (2017). Calcaneal cysts and lipomas: A common pathogenesis?. Skelet. Radiol..

[11] Pogoda P., Priemel M., Gebauer M., Rupprecht M., Adam G., Rueger J.M., Amling M. (2003). Kalkaneuszysten. Unfallchirurg.

[12] Levy D.M., Gross C.E., Garras D.N. (2015). Treatment of Unicameral Bone Cysts of the Calcaneus: A Systematic Review. J. Foot Ankle Surg..

[13] Sung A.D., Anderson M.E., Zurakowski D., Hornicek F.J., Gebhardt M.C. (2008). Unicameral Bone Cyst: A Retrospective Study of Three Surgical Treatments. Clin. Orthop. Relat. Res..

[14] Toepfer Keller S., Meester J.A. (2018). Unicameral bone cyst of the calcaneus. Orthopädische Unf. Prax.

[15] Toepfer A., Lenze U., Gerdesmeyer L., Pohlig F., Harrasser N. (2016). Endoscopic resection and allografting for benign osteolytic lesions of the calcaneus. Springerplus.

[16] Hoshi M., Iwai T., Oebisu N., Shimatani A., Takada N., Nakamura H. (2021). Pathological fracture of a solitary bone cyst in the calcaneus: A case series and literature review. Arch. Orthop. Trauma Surg..

[17] Pogoda P., Priemel M., Linhart W., Stork A., Adam G., Windolf J., Rueger J.M., Amling M. (2004). Clinical relevance of calcaneal bone cysts: A study of 50 cysts in 47 patients. Clin. Orthop. Relat. Res..

[18] Milgram J.W. (1988). Intraosseous lipomas. A clinicopathologic study of 66 cases. Clin. Orthop. Relat. Res..

[19] Radl R., Leithner A., Machacek F., Cetin E., Koehler W., Koppany B., Dominkus M., Windhager R. (2004). Intraosseous lipoma: Retrospective analysis of 29 patients. Int. Orthop..

[20] Alnooh A.M., Al Furaikh B.F., Alaithan A.M., Halawani A.K., Al-Khalifah M.F., Abu Zahirah M.O., Alhasani M.H., Asiri A.A., Alrashid A.H., Alfaqih E.H. (2022). Intraosseous Calcaneal Lipoma Misdiagnosed as Plantar Fasciitis: An Orthopedic Case From Family Practice. Cureus.

[21] Schuh A., Koehl P., Sesselmann S., Goyal T., Benditz A. (2022). Incidental intraosseous calcaneal lipoma in a patient suffering from plantarfasziitis. Georgian Med. News.

[22] Karthik K., Aarthi S. (2011). Intraosseous lipoma of the calcaneus mimicking plantar fascitis. Foot Ankle Surg. Off J. Eur. Soc. Foot Ankle Surg..

[23] Weger C., Frings A., Friesenbichler J., Grimer R., Andreou D., Machacek F., Pfeiffenberger K., Liegl-Atzwanger B., Tunn P.-U., Leithner A. (2013). Osteolytic lesions of the calcaneus: Results from a multicentre study. Int. Orthop..

[24] Toepfer A. (2018). Ossoscopy of benign osteolytic lesions of the calcaneus. Arthroskopie.

[25] Weinfeld G.D., Yu G.V., Good J.J. (2002). Intraosseous lipoma of the calcaneus: A review and report of four cases. J. Foot Ankle Surg. Off Publ. Am. Coll. Foot Ankle Surg..

[26] Murphey M.D., Carroll J.F., Flemming D.J., Pope T.L., Gannon F.H., Kransdorf M.J. (2004). From the archives of the AFIP: Benign musculoskeletal lipomatous lesions. Radiogr. A Rev. Publ. Radiol. Soc. N. Am. Inc..

[27] Ulucay C., Altintas F., Ozkan N.K., Inan M., Ugutmen E. (2009). Surgical treatment for calcaneal intraosseous lipomas. Foot.

[28] Campbell R.S.D., Grainger A.J., Mangham D.C., Beggs I., Teh J., Davies A.M. (2003). Intraosseous lipoma: Report of 35 new cases and a review of the literature. Skelet. Radiol..

[29] Milgram J.W. (1988). Intraosseous lipomas: Radiologic and pathologic manifestations. Radiology.

[30] Malghem J., Lecouvet F., Omoumi P., Berg B.V. (2021). Intraosseous lipomas originating from simple bone cysts. Skelet. Radiol..

[31] Sirry A. (1951). The Pseudo-Cystic Triangle in the Normal OS Calcis. Acta Radiol..

[32] Freyschmidt J., Brossmann J., Sternberg A., Wiens J. (2003). Borderlands of Normal and Early Pathological Findings in Skeletal Radiography.

[33] Smith R.W., Smith C.F. (1974). Solitary unicameral bone cyst of the calcaneus. A review of twenty cases. J. Bone Jt. Surg. Am..

[34] Campanacci M., Campanacci M. (1999). Simple bone cysts. Bone Soft Tissue Tumors.

[35] Copleman B., Vidoli M.F., Crimmings F.J. (1946). Solitary cyst of the calcaneus. Radiology.

[36] Van Linthoudt D., Lagier R. (1978). Calcaneal Cysts:A Radiological and Anatomico-Pathological Study. Acta Orthop..

[37] Elias I., Zoga A.C., Raikin S.M., Schweitzer M.E., Morrison W.B. (2007). Incidence and Morphologic Characteristics of Benign Calcaneal Cystic Lesions on MRI. Foot Ankle Int..

[38] Hoshi M., Oebisu N., Iwai T., Shimatani A., Takada N., Aono M., Ieguchi M., Takami M., Nakamura H. (2019). Possible pathogenesis of calcaneal bone cysts. Arch. Orthop. Trauma Surg..

[39] Cohen J. (1970). Etiology of simple bone cyst. J. Bone Jt. Surg. Am..

[40] Cohen J. (1960). Simple bone cysts. Studies of cyst fluid in six cases with a theory of pathogenesis. J. Bone Jt. Surg. Am..

[41] Andermahr J., Jubel A., Prokop A., Kasper H.-U., Eisner A., Rehm K.E., Koebke J. (2004). The calcaneal cyst—Pathogenesis and intraosseous vascularization of the calcaneus. Fuß Sprunggelenk.

[42] Chigira M. (1983). The Aetiology and Treatment of Simple Bone Cysts. JBJS Br..

[43] Toepfer A., Harrasser N., Lenze U., Liska F., Mühlhofer H., Von Eisenhart-Rothe R., Banke I.J. (2015). Bilateral diaphyseal bone cysts of the tibia mimicking shin splints in a young professional athlete—A case report and depiction of a less-invasive surgical technique. BMC Musculoskelet. Disord..

[44] Hatori M., Hosaka M., Ehara S., Kokubun S. (2001). Imaging features of intraosseous lipomas of the calcaneus. Arch. Orthop. Trauma Surg..

[45] Hart J.A. (1973). Intraosseous lipoma. J. Bone Jt. Surg. Br..

[46] Richardson A.A., Erdmann B.B., Beier-Hanratty S., Lautz D., Jacobs P.M., Julsrud M.E., Ringstrom J.B. (1995). Magnetic resonance imagery of a calcaneal lipoma. J. Am. Podiatr. Med. Assoc..

[47] Lagier R. (1980). Case report 128: Lipoma of the calcaneus with bone infarct. Skeletal Radiol.

[48] Lagier R. (1985). Fibular lipoma with areas of bone infarct calcification. Eur. J. Radiol..

[49] Barker G.R., Sloan P. (1986). Intraosseous lipomas: Clinical features of a mandibular case with possible aetiology. Br. J. Oral. Maxillofac. Surg..

[50] Tins B.J., Berkowitz Y.J., Konala P., Davies M., Cassar-Pullicino V.N., Lalam R., Cool P. (2021). Intraosseous lipomas originating from simple bone cysts. Skelet. Radiol..

[51] Neer C.S., Francis K.C., Marcove R.C., Terz J., Carbonara P.N. (1966). Treatment of unicameral bone cyst. A follow-up study of one hundred seventy-five cases. J. Bone Jt. Surg. Am..

[52] Hou H.-Y., Wu K., Wang C.-T., Chang S.-M., Lin W.-H., Yang R.-S. (2010). Treatment of unicameral bone cyst: A comparative study of selected techniques. J. Bone Jt. Surg..

[53] Aiba H., Kobayashi M., Waguri-Nagaya Y., Goto H., Mizutani J., Yamada S., Okamoto H., Nozaki M., Mitsui H., Miwa S. (2018). Treatment of aneurysmal bone cysts using endoscopic curettage. BMC Musculoskelet. Disord..

[54] Dindo D., Demartines N., Clavien P.-A. (2004). Classification of Surgical Complications: A new proposal with evaluation in a cohort of 6336 patients and results of a survey. Ann. Surg..

[55] Clavien P.A., Barkun J., de Oliveira M.L., Vauthey J.N., Dindo D., Schulick R.D., de Santibañes E., Pekolj J., Slankamenac K., Bassi C. (2009). The clavien-dindo classification of surgical complications: Five-year experience. Ann. Surg..

[56] Dodwell E.R., Pathy R., Widmann R.F., Green D.W., Scher D.M., Blanco J.S., Doyle S.M., Daluiski A., Sink E.L. (2018). Reliability of the Modified Clavien-Dindo-Sink Complication Classification System in Pediatric Orthopaedic Surgery. JBJS Open Access.

[57] Müller A.M., Toepfer A., Harrasser N., Haller B., Walther M., von Eisenhart-Rothe R., Gemperlein K., Bergmann K., Bradaric C., Laugwitz K.-L. (2019). Significant prevalence of peripheral artery disease in patients with disturbed wound healing following elective foot and ankle surgery: Results from the ABI-PRIORY (ABI as a PRedictor of Impaired wound healing after ORthopedic surgerY) trial. Vasc. Med..

[58] Toepfer A., Lenze U., Harrasser N. (2016). Calcaneal Ossoscopy. Arthrosc. Tech..

[59] Jones K.B., DeYoung B.R., Morcuende J.A., Buckwalter J.A. (2006). Ethanol as a local adjuvant for giant cell tumor of bone. Iowa Orthop. J..

[60] Solooki S., Keikha Y., Vosoughi A.R. (2017). Can ethanol be used as an adjuvant to extended curettage in order to reduce the recurrence rate of aneurysmal bone cyst?. Rev. Bras. Ortop..

[61] Lin W.H., Lan T.Y., Chen C.Y., Wu K., Yang R.S. (2011). Similar local control between phenol- and ethanol-treated giant cell tumors of bone. Clin. Orthop. Relat. Res..

[62] Anwander H., Hug U., Fuchs B. (2019). Excessive white drainage after Cerament filling and curettage of an aneurysmal bone cyst at the distal radius. Swiss Med. Wkly..

[63] Gärds G. (2018). Cerament Bone Void Filler Customer Information. https://www.google.de/url?sa=t&rct=j&q=&esrc=s&source=web&cd=&ved=2ahUKEwjj18u8h5D-AhWhTOUKHdjXBYMQFnoECAsQAQ&url=https%3A%2F%2Ffsca.swissmedic.ch%2Fmep%2Fapi%2Fpublications%2FVk_20181116_19%2Fdocuments%2F0&usg=AOvVaw2qtylSY4ZgAYSry2MColUv.

[64] Kawaguchi M., Kato H., Miyazaki T., Nagano A., Matsuo M. (2022). Imaging Findings of Calcaneal Cyst and Lipoma: Can Intraosseous Cyst Changes into Lipoma with Advancing Age?. J. Comput. Assist. Tomogr..

[65] Ma H., Shi Y., Zhang W., Liu F., Han Y., Yang M. (2021). Open Curettage with Bone Augmentation for Symptomatic Tumors and Tumor-like Lesions of Calcaneus: A Comparison of Bioactive Glass Versus Allogeneic Bone. J. Foot Ankle Surg..

[66] Karr J.C. (2019). Calcium sulfate/calcium phosphate bone void filler in the treatment of bilateral adolescent unicameral calcaneal bone cysts: 36-month follow-up. J. Am. Podiatr. Med. Assoc..

[67] Aycan O.E., Keskin A., Sökücü S., Özer D., Kabukçuoğlu F., Kabukçuoğlu Y.S. (2017). Surgical Treatment of Confirmed Intraosseous Lipoma of the Calcaneus: A Case Series. J. Foot Ankle Surg..

[68] Yildirim C., Akmaz I., Sahin O., Keklikci K. (2011). Simple calcaneal bone cysts: A pilot study comparing open versus endoscopic curettage and grafting. J. Bone Jt. Surg. Ser. B.

[69] Bonnel F., Canovas F., Faure P. (1999). Treatment of a simple bone cyst of the calcaneus by endoscopic curettage with cancellous bone injection. Acta Orthop. Belg..

[70] Mainard D., Galois L. (2006). Treatment of a Solitary Calcaneal Cyst with Endoscopic Curettage and Percutaneous Injection of Calcium Phosphate Cement. J. Foot Ankle Surg..

[71] Alvarez R.G., Arnold J.M. (2007). Technical Tip: Arthroscopic Assistance in Minimally Invasive Curettage and Bone Grafting of a Calcaneal Unicameral Bone Cyst. Foot Ankle Int..

[72] Roth S., Sestan B., Madarevic T., Gulan G., Gruber B., Miletic D. (2010). Endoscopic assistance in the treatment of calcaneal and humeral juvenile bone cysts. J. Orthop. Sci..

[73] Innami K., Takao M., Miyamoto W., Abe S., Nishi H., Matsushita T. (2011). Endoscopic Surgery for Young Athletes with Symptomatic Unicameral Bone Cyst of the Calcaneus. Am. J. Sports Med..

[74] Yıldırım C., Mahirog M., Kuskuku M., Akmaz I., Keklikci K. (2010). The Journal of Foot & Ankle Surgery Treatment of a Unicameral Bone Cyst of Calcaneus with Endoscopic Curettage and Percutaneous Filling with Corticocancellous *Allograft*. J. Foot Ankle Surg..

[75] Nishimura A., Matsumine A., Kato K., Aasanuma K., Nakamura T., Fukuda A., Sudo A. (2016). Endoscopic Versus Open Surgery for Calcaneal Bone Cysts: A Preliminary Report. J. Foot Ankle Surg..

[76] Stoica I.C., Pop D.M., Grosu F. (2017). Unicameral bone cyst of the calcaneus—Minimally invasive endoscopic surgical treatment. Case report. Rom. J. Morphol. Embryol..

[77] Farouk H.A., Saladin M., Abu Senna W., Ebeid W. (2018). All-endoscopic management of benign bone lesions; a case series of 26 cases with minimum of 2 years follow-up. Sicot-J.

[78] Megremis P.K., Orestis P., Megremis O.P. (2021). Endoscopic High-speed Curettage and Bone Grafting of Unicameral Bone Cyst in Young Children. Tech. Foot Ankle Surg..

[79] Yi Y., Lee J.S., Kim J., Jin S.Y., Won S.H., Cho J., Chun D.-I. (2021). Efficacy of lesion specific portals in endoscopic treatment of calcaneal bone cyst: A case report and literature review. Medicina.

[80] Choi Y., Kwak J.M., Chung S.H., Jung G.H., Kim J.D. (2014). Tumor treated by endoscopy. Clin. Orthop. Surg..

[81] Bishay S.N.G. (2015). Curettage without bone grafting for a simple bone cyst in the capitate. J. Orthop. Surg..

[82] Salunke A.A. (2016). Curettage without Bone Grafting for a Simple Bone Cyst in the Capitate. J. Orthop. Surg..

[83] Hirata M., Kusuzaki K., Hirasawa Y. (2001). Eleven cases of intraosseous lipoma of the calcaneus. Anticancer Res..

[84] Huch K., Werner M., Puhl W., Delling G. (2004). Die Kalkaneuszyste: Eine klassische solitäre Knochenzyste?. Z. Orthop. Ihre Grenzgeb..

[85] Howe B.M., Powell G.M., Ringler M.D., Iii N.S.T., Broski S.M., Turner N.S. (2018). Intraosseous “Lipoma” of the Calcaneus Developing in an Intraosseous Ganglion Cyst. J. Radiol. Case Rep..

